# Lessons Learned From the Implementation of an Integrated Health and Social Care Child and Family Hub – a Case Study

**DOI:** 10.5334/ijic.8631

**Published:** 2024-11-15

**Authors:** Sarah Loveday, Natalie White, Leanne Constable, Anthony Gates, Lena Sanci, Sharon Goldfeld, Harriet Hiscock

**Affiliations:** 1 Department of Paediatrics, University of Melbourne, Australia; 2 Health Services, Murdoch Children’s Research Institute, Melbourne, Victoria, Australia; 3 Policy and Equity, Murdoch Children’s Research Institute, Melbourne, Victoria, Australia; 4 Centre for Community Child Health, The Royal Children’s Hospital, Melbourne, Victoria, Australia; 5 Parenting Research Centre, Australia; 6 Department of General Practice, University of Melbourne, Australia

**Keywords:** childhood adversity, integrated care, rapid qualitative ethnography, practice change

## Abstract

**Introduction:**

Childhood adversity is associated with poor physical and mental health outcomes across the lifespan. Integration of health and social care may provide a solution to childhood adversity through practices of better detection and response. There is growing interest in the creation of child and family hubs that integrate health and social care but little literature that describes the development process.

**Description:**

We aimed to evaluate and describe the implementation of a co-designed health and social care child and family hub in Victoria, Australia. Rapid ethnographic methodology was used to iterate the hub components. Practitioners and researchers co-created solutions to barriers identified during implementation.

**Discussion:**

There were five key learnings: (i) Practice change takes time and intensive coaching, (ii) Lived experience is a powerful motivator for practice change, (iii) Integration of services requires more than co-location to break down silos, (iv) Reflective practice is a key driver of practice change, and (v) Using real time data enabled rapid implementation change and directly informed the development of solutions.

**Conclusions:**

Maintaining and developing practice change during implementation requires time and access to a broad range of data to enable iteration and the development of solutions.

## Introduction

Childhood adversity has been recognised as an urgent public health challenge due to the association between experiencing adversity in childhood and poor physical and mental health outcomes in later life [1]. Childhood adversity encompasses a range of experiences such as parental drug and alcohol abuse, parental mental health challenges, child abuse and neglect, family or community violence, food and housing insecurity, and bullying [1,2]. Childhood adversity has a strong correlation with poor mental health outcomes in later life with 30% of all mental health issues attributed to childhood adversity including anxiety, depression and suicidal attempts [3]. Although addressing childhood adversity early has the potential to improve outcomes children experiencing adversity often do not have access to help in a timely and effective manner [4]. Accessing help for childhood adversity is effortful and has an emotional toll on caregivers which is compounded by the fragmentation across health and social care systems [5].

Integrated care models have the potential to address the fragmentation of the health and social care systems and to improve access to services for families experiencing adversity [6]. Integrated care initiatives can increase uptake and improve engagement with child health services and are acceptable to families through providing holistic care and coordination across health, social and education systems [7,8]. There has been increasing interest in integrated community healthcare hubs as a “one-stop” solution to providing a range of services for individuals with complex health and social needs. While there is no single definition of a hub, it is often used to describe a centralised location that provides services that are co-located helping improve linkages to community agencies [9]. Hubs have been shown to improve access to services for families who are experiencing increased social needs [10]. Despite the rapid development of community hubs across Australia and around the world, little is published about the process of their implementation. There is a need for publication of papers describing the process and challenges of implementation to enable broader learning. Understanding what influences successful implementation and the contextual factors which impact practice change across different projects is essential to ensure that appropriate implementation strategies are used and evaluated.

Qualitative methodologies play a critical role in answering how and why changes happen or don’t happen, and identify contextual factors that influence this change [11,12]. However, there are some unique challenges in conducting qualitative research within an implementation framework due to the need for rapid results, multi-level stakeholder perspectives and the different contextual changes over time [12]. There has been growing interest in the development of qualitative methodologies that are able to overcome these challenges while still maintaining scientific rigour, including rapid qualitative ethnography [13,14,15], rapid assessment procedure (RAP) [16] and rapid analysis techniques [17]. However more papers need to be published demonstrating how these methods can be employed and to share the learning across different disciplines. To date there have been no papers published using rapid qualitative methodologies in integrated child and family health initiatives.

This paper describes the implementation of an integrated health and social care child and family hub and highlights the key learnings from the implementation. We aim to describe the strategies and iteration processes used to overcome barriers to implementation and support practice change. We use rapid ethnographic methodology to repeatedly identify and address barriers to implementation.

## Description of the Child and Family Hub

### Setting

The Child and Family Hub (CFH) was part of broader research project which aimed to co-design, test and evaluate two integrated health and social care hub models to improve the identification and response to adversity for children aged 0–8 years and their families in the states of Victoria and New South Wales, Australia [9]. This paper describes the implementation of the CFH at IPC Health, Wyndham Vale, Victoria. Wyndham is a culturally diverse local government area in the western suburbs of Greater Melbourne which has a rapidly growing population and has higher rates of unemployment, housing stress and social isolation compared to greater Melbourne [18]. IPC Health is a community health organisation that provides a diverse range of community services across six sites in Western Melbourne for the local population from children to elderly.

### Design of the Child and Family Hub

**Figure 1:**
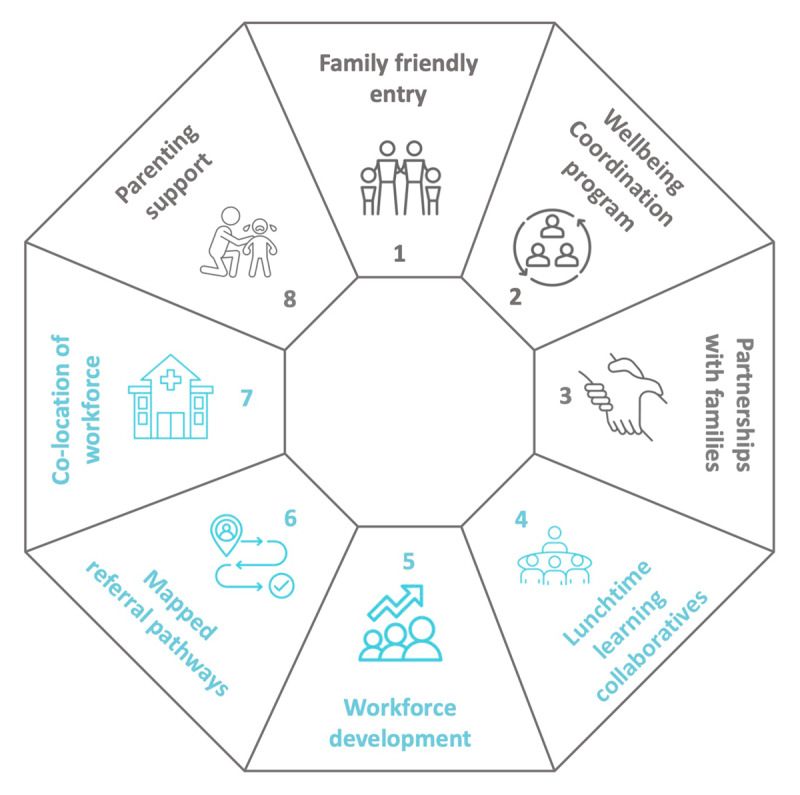
Core Components of the co-designed Child and Family Hub. The family facing components are denoted in grey and practitioner components in blue.

The CFH was designed through a co-design process involving community members, families with lived experience and practitioners across health and social care from IPC Health and community agencies in the local area [19]. The aim of the CFH was to improve children’s mental health by enhancing the detection and response to adversity. Initially a CFH model was proposed with six core components [20] which was further developed to eight core components as illustrated in Figure 1.

Four core components were ‘family facing’ and centred around the family journey through the CFH. These components were codesigned over a 10-week period by community members (n = 2 parents) and practitioners (n = 6 across health and social care) which has been described elsewhere [19]. The family friendly entry is a ‘no wrong door’ approach with caregivers asked about adversity and supported regardless of whom they saw at the CFH. The wellbeing coordination program was a mix of patient navigation and social prescribing which aimed to support caregivers to identify the holistic needs of their child and then help caregivers navigate services and social supports in the community e.g. drug and alcohol supports, housing, mental health support. Partnerships with families involved the intentional development and strengthening of connections between the CFH and the community through community connect days in which community members could ‘drop in’ without an appointment and talk to Hub practitioners i.e. wellbeing coordinator and legal practitioner. Parenting support aimed to support parents through light touch parent coaching delivered by CFH practitioners during clinical encounters. The light touch parent coaching was developed by a member of the research team (AG) using a single session approach [21].

The other four components were ‘practitioner facing’ and were designed to improve practitioner confidence and competence to identify and respond to childhood adversity. This included workforce development and training to ask difficult questions using the principles of the Family Partnership Model [22] as well as using the Parent Engagement Resource (PER) which is a tool designed to support practitioners to directly ask about adversity [23]. Monthly ‘lunchtime learning collaboratives’ had a participatory action framework wherein practitioners across health and social care identified barriers to implementation of CFH components and co-designed solutions. These meetings were designed to embed training and develop collaborative practice. To ensure that all practitioners had the opportunity to share ideas and to overcome potential power differentials in the group, researchers used different techniques to gather feedback including anonymous written feedback as well as invited feedback during group discussion. Learning collaborative meetings were facilitated by a member of the implementation team (AG). Mapped referral pathways were developed to improve practitioner confidence to respond to adversity by providing a community directory of services for different types of adversity. Finally, co-location of the workforce was an essential part of building collaborative practice by enabling the development of relationships and warm referrals across a range of services and practitioners.

**Figure 2:**
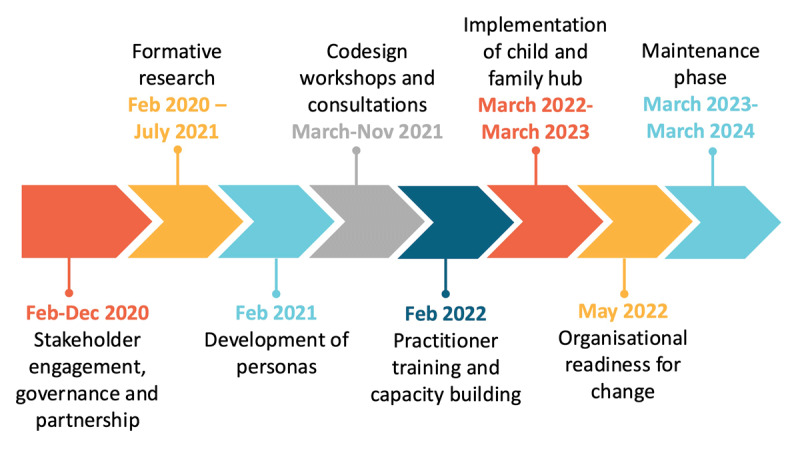
Timeline of Child and Family Hub from development to implementation.

The timeline for the broader research project is seen in Figure 2. The initial stages of this project have been described elsewhere [19]. This paper describes the iterations to the CFH during implementation with a focus on the practitioner components as described above.

### Practitioners in the Child and Family Hub

Following the codesign and consultation phase, practitioners who worked across health and social care were approached to take part in the implementation of the CFH. Health practitioners (general practitioners, paediatricians, allied health practitioners and nurses) all worked at IPC Health prior to implementation. Two practitioners declined to participate due to work pressures (general practitioner and community nurse). The social care practitioners (financial counsellor, wellbeing co-ordinator and lawyers) were all new roles as part of the development of the CFH. Three lawyers came from two separate legal organisations and provided services in-kind to the CFH. The financial counsellor also provided services in-kind as IPC Health was restricted to providing gambling support services prior to implementation of the CFH.

At the start of implementation there were 18 practitioners across health and social care in the CFH. However, there were several changes that happened over the 12-month implementation phase. Three practitioners (nurse, financial counsellor and paediatric fellow) left their roles in the CFH due to a change in employment (at 4 months, 10 months, and 11 months, respectively). Two practitioners joined the CFH (practice nurse and maternal child health nurse) after 9 months. In addition, three new lawyers integrated into the CFH at 9 months.

Practitioner written consent and demographic information were collected prior to the implementation of the Child and Family Hub through the online database REDcap. [See supplementary material for demographic characteristics of practitioners] Due to the high potential of identification practitioners are referred to by number rather than professional disciple.

### Practitioner participation requirements

Participation in the CFH required a commitment from practitioners to attend 1 day of training at the commencement of the CFH to improve their competence and comfort to directly ask about adversity and attendance at monthly learning collaborative meetings for the 12 months of the implementation phase. In addition, practitioners were required to attend a half day workshop on developing integrated practice and psychological safety. Attendance at monthly meetings was recorded as a marker of practitioner engagement.

### Evaluation of implementation

#### Implementation team

A subgroup of the broader research team formed the implementation team (SL, NW, AG, LC). They were responsible for engaging with practitioners and managers and ensuring the implementation of the CFH components. The implementation team was co-located at the CFH site in Wyndham vale.

#### Rapid ethnography methodology

It was initially planned that the methodology of Plan-Do-Study-Act cycles [24] would be utilised to address barriers that impeded practitioners from identifying and responding to adversity however, it was quickly determined that practitioners were not able to collect quantitative data in a way that could be meaningfully used, therefore rapid ethnography methodology was employed to guide iterative cycles as this data was already being collected.

Ethnography is the study of social interactions, behaviours and perceptions that occur within groups. The aim is to use naturalistic observations, interviews, and relationships with participants to develop insights into people’s views and actions thus generating emic perspectives. While traditional ethnography methodology is time consuming and labour intensive, rapid qualitative ethnography has been successfully used in a range of implementation studies [13,14,15]. Rapid ethnography utilises mixed methods data with the inclusion of quantitative process data in addition to traditional qualitative observations and interviews and applies a team approach to analysis. There is a focus on participation of the study population and iterative cycles of data collection and analysis which lends itself to use in project implementation [16].

Multiple sources of data in keeping with rapid ethnographic methodology were used to guide implementation as outlined in Table 1. The implementation team were embedded at the CFH which enabled regular conversations with practitioners helping to uncover implementation issues as they arose and to develop an emic understanding of the barriers to change in a community health setting. Observational data (field notes) and transcripts were analysed using reflective thematic analysis as described by Braun and Clarke [25] by the implementation team each month to determine the main themes and barriers to implementation. Line by line coding of transcripts and field notes were used to develop themes. Discrepancies in identifying themes was resolved through discussion. Process data i.e. number of referrals to legal support, wellbeing co-ordinator and financial counsellor, was triangulated with qualitative data to understand practitioner confidence to ask and respond to adversity. Topics for monthly meetings were developed by implementation team based on themes from qualitative analysis as well as through feedback by practitioners.

**Table 1:** Data Collection During Implementation.


DATA TYPE	TIME POINT	COLLECTION METHOD	PURPOSE OF DATA	RESEARCHER

Observation	During clinical encounters at baseline and at 6 months	Notes detailing how practitioners asked about adversity and what types of adversity discussed during clinical encounters	To determine if there was any observable change in practitioners directly asking about adversity	SL

Observation	During initial practitioner training	Field notes from conversations with practitioners during training	To determine how comfortable practitioners were at baseline to address adversity	SL, NW, LC

Observation	During monthly learning collaboratives	Field notes from conversations with practitioners during monthly meetings	To identify barriers and facilitators of CFH implementation	SL, NW

Observation	Throughout implementation	Field notes from conversations and emails from practitioners	To identify barriers and facilitators of CFH implementation	SL, NW

Transcripts	Monthly learning collaboratives	Direct quotes from practitioners	To identify barriers and facilitators of CFH implementation	SL, NW

Process Data	Each month throughout implementation	Number of referrals received by each Hub service collected monthly	To show changes in referral patterns over the 12 months	NW


#### Reflexivity

The research team brought different perspectives to the analysis with a paediatrician (SL), a senior research officer (NW), a lived experience peer researcher (LC) and a psychologist with expertise in mental health and parenting support (AG). The rest of the research team were medical doctors (HH, LS, SG). SL is an experienced qualitative researcher and led the qualitative analysis. The implementation team bought both professional and lived experience to their analysis.

### Ethics

The study was conducted in line with the National Statement on Ethical Conduct in Human Research [26]. Ethical approval was granted by The Royal Children’s Hospital Human Research Ethics Committee (HREC # 62866.7).

## Implementation of the Child and Family Hub: Results

### Participation

Attendance of health and social care practitioners at monthly learning collaboratives was variable with half the 18 practitioners attending for 75% or more of meetings (n = 9, 50%), some attending between 50% –75% (n = 5, 28%) and others attending less than 33% of the time (n = 4, 22%). There was no difference between health and social care practitioners in attendance with both groups having 50% of practitioners attending for at least 75% of learning collaboratives. General Practitioners (GPs) were paid to attend meetings as the Australian fee-for-service model precluded monthly involvement due to loss of income for practitioners. This supported GPs to attend meetings and helped to foster relationships across primary care and other health practitioners as well as health and social care practitioners.

### Iteration during Implementation

#### Beginning of Implementation (months 1–3)

The main themes which were identified as barriers or facilitators of implementation are highlighted in Table 2. Early in the implementation of the Hub, practitioners were uncertain about their role within the CFH and what the CFH was. They saw the CFH as a place to refer patients rather than as a mechanism to help change the way they were practicing. Practitioners were quick to recognise the benefits of working in a wider team but lacked confidence to directly ask about adversity. Interestingly, both health care practitioners and social care practitioners were reluctant to directly ask about adversity.

**Table 2:** Topics and themes of monthly learning collaboratives.


MONTH	TOPIC DISCUSSION AT LEARNING COLLABORATIVE	THEMES IDENTIFIED EACH MONTH WITH SUPPORTIVE QUOTES

Month 1	**Working together** Reflection on training and establishing CFH	**Theme: Uncertainty** **Uncertainty in how to approach adversity**. Practitioners were unsure how to ask about adversity “*Do I push further?*” (P1) not wanting to duplicate asking about adversity *“not overstepping our practice”* (P6). **Uncertainty in understanding the CFH**. Seeing the Hub as an external service rather than integrated care *“I have many families suitable for the Hub”* (P1)

Month 2	**Legal Support** How the lawyers can help to address adversity	**Theme: Building relationships in the CFH**. Starting to form relationships across practitioners *“great to have communication across the team”* (P3), to *“know your face”* (P14) **Theme: Fear of damaging relationship**. Afraid to talk to a family about family violence for fear of damaging relationship “*so careful about that [asking about family violence] as there is already so much guilt for a mother who stays in a violent relationship”* (P1)

Month 3	**Reframing parenting as a solution** Introducing role of mental health expert in CFH	**Theme: Permission to directly ask** Practitioners finding it difficult to directly ask especially families they know well *“I feel uncomfortable asking about financial questions because I don’t have this issue”* (P1) *“this is not relevant to what I am doing here so I leave it”* (P12) *“the box goes unchecked”* (P4)

Month 4	**Permission Giving** Lived experience researcher shared her thoughts on being asked about adversity	**Theme: Barriers to engagement with parents** Practitioners reflected on barriers to engagement with parents and the need to build trust. Families were seen as *“hard to work with”* (P12) but that *“it doesn’t have to be as hard”* (P5) **Theme: What is the Hub?** Practitioners were still confused about the CFH and saw this as a referral to the Wellbeing Coordinator *“I referred to [WBC] as a starting point”*(P2)

Month 5	**Micro-coaching parents** Supporting practitioners to use all opportunities to provide coaching to parents	**Theme: Parent engagement through building trust** Practitioners had difficulties with engaging families, and it took time to find out about adversity *“[legal issues] came out after a few conversations…found out different bits and pieces as they trust you”* (P20) parents seen as *“not willing to engage”* (P9)

Month 6	**Reflective Practice** Practitioners given exercise to help them reflect on their own practice change.	**Theme: Importance of being in a team** Practitioners discussed the best things about being in the CFH as being in a team *“connecting with practitioners that we ordinarily wouldn’t connect with”* (P7), *“opportunity to network, to meet regularly, to get to know the team”* (P8) **Theme: Feeling ill-equipped** Practitioners reflected on feeling *“out of depth, out of my scope so I found it challenging”* (17) and *“if I had to do it again, I would ask questions differently.”* (P13) Barriers in language used.

Month 7	**Family Violence** Aim to improve practitioner comfort to directly ask about family violence	**Theme: Opening a can of worms** Fear of directly asking about family violence. *“putting clients in further danger”* (P13) *“I’ve asked then what”* (P12) *“asking may affect the relationship I have with the person, they may be offended”* (P17) *“You might need to open a can of worms”*(P16). Challenges in getting client alone to ask *“how to deal and treat it with a child in the room”* (P2)

Month 8	**Perinatal Mental Health** Lived experience of getting help for mental health issues	**Theme: Power of asking about adversity** Practitioners recognising the value of asking about adversity *“you don’t lose anything by asking and most people will be glad that you asked even if it is not true at that moment”*(P1) **Theme: Overcoming stigma of mental health** Practitioners discussed the challenges to overcome stigma when asking about mental health *“it can be difficult sometimes…it might affect future income protection”* (P4)

Month 9	**Reflections on Learnings** Practitioner reflection on what they had learnt so far in CFH	**Theme: It’s Ok to ask about adversity** Practitioners recognised that they did not have to solve all the problems when they asked about adversity, *“I have changed the way I think about it to not trying to solve the problem…I feel more comfortable doing the holding now”*(P1) Positive experiences were encouraging *“So I feel that goes to what you have been saying all along is that people don’t mind being asked [about adversity] and people don’t mind being approached and it can be a relief”* P20.

Month 10	**The words we use** Improving confidence to directly ask with focus on the language used	**Theme: Time poor** Practitioners discussed that they found it difficult to identify adversity because they did not *“have time”* (P3) *“so much adversity…hard to know where to start…we are completely rushed”* (P2) **Theme: Families disclose adversity** Practitioners felt that families were disclosing so much adversity that they did not need to ask directly *“they will burst into tears, and they will blurt out or vomit everything”*(P21)

Month 11	**The next step in practice** Practitioners planning the next step in their own practice change	**Theme: Evolution of practice** Practitioners recognised the changes they had made in their practice and how asking about adversity was beneficial. *“Can I just say I love how to pose these questions has evolved from the beginning, because they’re so much more approachable”* (P1) Practitioners were more comfortable to take the next step in practice *“taking more initiative to ask questions”* (P5)

Month 12	**Housing support** Education about options for housing support	**Theme: Overwhelmed/You are asking too much of me** Practitioners discussed feeling *“completely overwhelmed”* (P21) and under *“constant pressure”* (P1) trying to address adversity while being time poor and knowing “*how you do deal with it once they [families] tell you all of these things”*(P21)


Observational data at baseline and at the time of initial training, indicated that practitioners were not comfortable to directly ask about adversity and were going to need more than training to effect change in practice. One of the implementation team (AG) was the facilitator of the monthly meetings and provided coaching and support to practitioners to change practice. Monthly meetings were recognised as too infrequent to bring about practice change so brief coaching emails to act as ‘nudges’, were sent bi-weekly to expand on themes and topics identified during implementation. [see supplementary material for example] AG also offered to meet with individual practitioners to provide coaching and support between meetings. Three practitioners took up this offer and met with AG several times over the 12 months.

**Figure 3:**
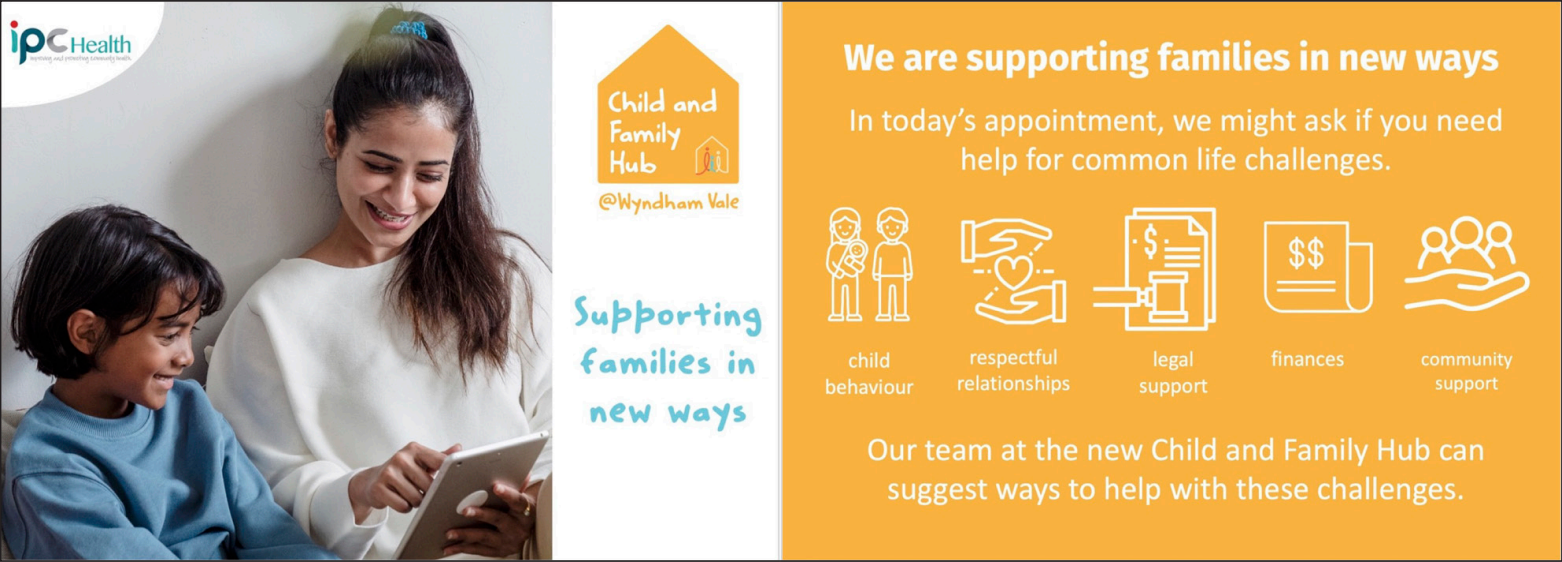
Postcards to address permission giving.

During the first three months of implementation the barriers to practitioners changing practice and asking about adversity included a fear of damaging relationships with families and not feeling like they had permission to ask, *“we don’t think that families want to be asked multiple times”* (Practitioner 9). In response to these barriers, postcards (Figure 3) were co-designed with practitioners and the implementation team to highlight the different supports available in the CFH. Through co-design with community, families stated that the preferred term to use when engaging with community was ‘life challenges’ and not ‘adversity’. The language used on the postcard reflects this learning. Postcards were given out by reception staff when caregivers presented for an appointment so that they could expect to be asked about adversity during their consultation with Hub practitioners. Practitioners reported that the postcards gave them more confidence to ask families about adversity during consultations although this was not witnessed in observations.

In addition to codesign of postcards, our lived experience researcher (LC) spoke at a monthly learning collaborative. She asked practitioners to be curious about adversity and to not underestimate the difference they can make to a family’s life by asking about adversity. This was a turning point for some practitioners and improved their confidence to start asking. We had further lived experience involvement at the monthly meeting in month 8 which reinforced for practitioners the benefits of asking caregivers about adversity and how to approach families with curiosity.

#### Middle Phase of Implementation (months 4–8)

In the middle phase of implementation there was a repeated theme of practitioners finding it difficult to engage with families and families were seen as *“hard to work with*” and *“not willing to engage”*. Practitioners were coached and encouraged to use motivational interviewing techniques (AG). Brief parenting handouts [see supplementary material for example] were also codesigned with practitioners to use with parents to help with engagement, which were reported to be helpful by parents. We recognised the importance of supporting practitioners to think about the differences they could make with families through small interventions, advice, and information, while families were waiting for external services.

Alongside these strategies, different ways of asking about adversity were workshopped with practitioners; this included introducing them to different tools that help identify adversity, i.e., the WECare tool [27], the PANDA toolbox [28], and questions based on a single session approach [21]. Practitioners reported that the PER tool, which was part of the initial training, was too long to use in their clinical encounters and the language was too complex for families as a high proportion were culturally and linguistically diverse. Practitioners most resonated with using different questions from different tools which increased their level of comfort with the language and approach. This combination of tools was then used for those *new* practitioners joining the CFH in updated training.

From observations at 6 months, it was clear that practitioners were unsure how to make referrals across the CFH and were not using the hard copy community services directory. One practitioner (Practitioner 13) commented that the *“hardest thing is referring them on”*. Accessing a hard copy version of the directory was proving to be a barrier to connecting with services so it was subsequently adapted to a soft copy and hosted on the CFH Microsoft Teams site. The CFH specific referral forms for legal services, wellbeing coordination and financial counsellor were also changed to online fillable forms and posters encouraging referrals to legal support were placed around the building to improve uptake. Practitioner 5 reported that her confidence was improved by the poster prompts in the CFH, *“after these were stuck up [gesturing towards posters about legal service], I felt more confident”*. Following the changes to the referrals there was a small increase in referrals after 6-months as seen in the process data below.

#### End Phase of Implementation (months 9–12)

From 8 months practitioners started to recognise the power of identifying adversity and were able to recognise their own evolution of practice to feeling more comfortable to ask about adversity and not having to “*solve the problem”* that their clients may be presenting with in one consultation. Practitioners recognised that practice change involved effort *“You have to make yourself do it [ask about adversity] or you can let yourself just keep doing what you normally do”* (Practitioner 17). This recognition of effort was enhanced by participating in reflective practice exercises during learning collaboratives at 6, 9 and 12 months. Reflective practice was seen as *“helpful to acknowledge that it [asking about adversity] is hard”* (Practitioner 8) and practitioners valued the opportunity to gain skills in reflective practice with one practitioner commenting that *“I have never done this before although I know I should”* (Practitioner 24).

While practitioners started to report an increased confidence to identify and respond to adversity towards the end of the implementation (12 months), they also reported ongoing barriers to asking about adversity due to being time pressured and feeling overwhelmed by the *“constant pressure*” and need in the community. Practitioners were more aware of adversity at the end of implementation and were responding to adversity more readily however, were still observed to be directly asking about adversity infrequently. The level of discomfort at the end of the implementation phase may reflect the practitioner concern about having to continue the CFH without the support of the implementation team and the facilitation or coaching support. However, the practitioners found the monthly meetings to be helpful and these were continued in the maintenance phase of the CFH.

### Process Data

**Figure 4:**
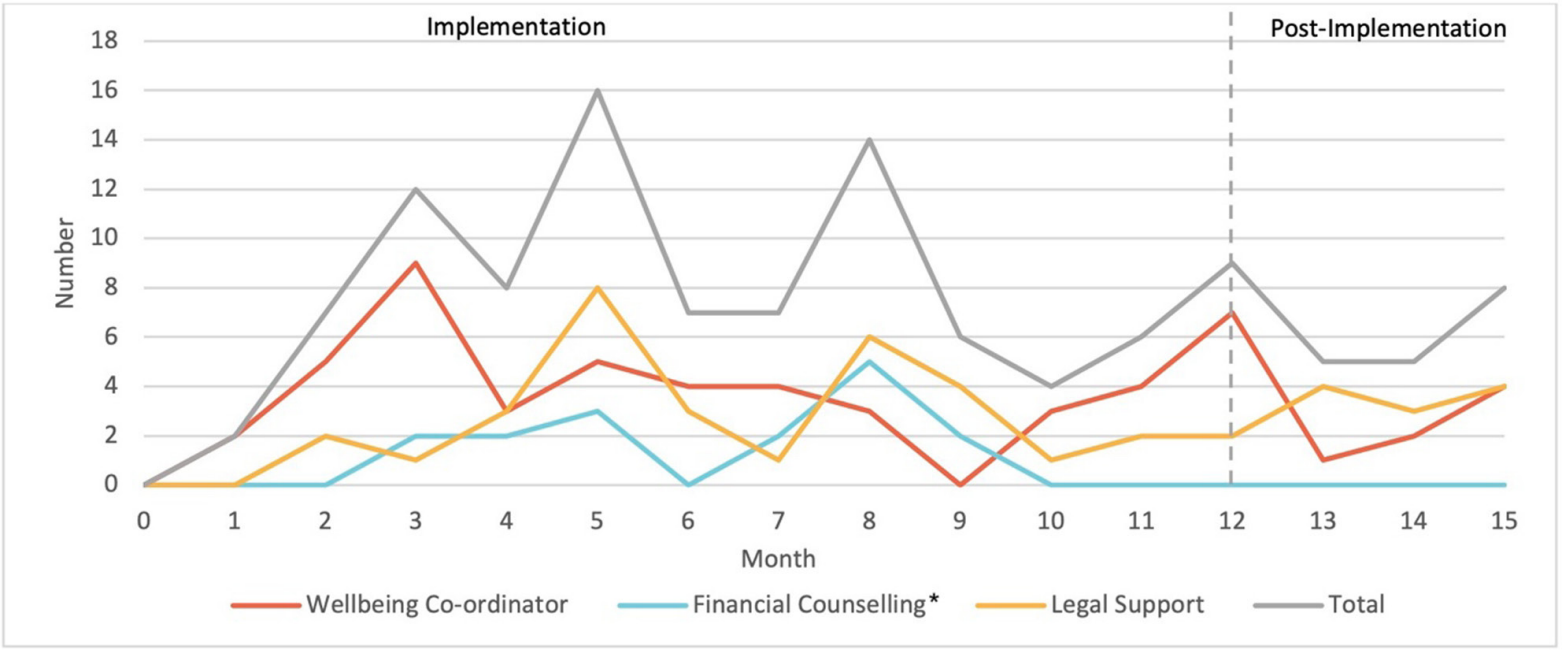
Referral patterns by month. *Financial counsellor left at 10 months.

The number of referrals made each month to legal support, wellbeing coordinator and financial counsellor are demonstrated in Figure 4. Process data was collected for the 12 months of implementation and for the first 3 months post implementation.

Over the first 12 months of the CFH there were small numbers of referrals made across all three areas with the smallest number of referrals to the financial counsellor. The referral rate dropped toward the end of implementation and stayed at this lower level in the immediate post implementation phase. There was a delay to referrals to both legal support and financial counselling as practitioners took time to feel comfortable to identify needs in these areas. Once the financial counsellor left the Hub, a new referral pathway to an external financial counsellor was developed. However, no data exists for the number of referrals made as it was not physically collocated in the CFH.

Referrals to the Wellbeing Coordinator did not demonstrate the initial delay however referrals dropped after the first three months. Practitioners were initially excited about the service but found the entry criteria restrictions such as current child protection involvement impacted on referral patterns.

Interestingly there were two large peaks in referrals at 5 months and 8 months which may corresponded to the two learning collaborative meetings that had in-session talks with people with lived experience of adversity. From observations, practitioners were motivated to identify adversity after these sessions as they recognised the importance of asking and were reassured that families were happy to be asked. This was reinforced with positive experiences of asking about adversity as is demonstrated by a quote from a practitioner *“As she [client] left she said I’m really glad you approached me, I feel relieved that I know that these supports are available. So, I feel that goes to what you have been saying all along is that people don’t mind being asked [about adversity] and people don’t mind being approached and it can be a relief”* (Practitioner 20).

### Organisation Readiness for Change

**Figure 5:**
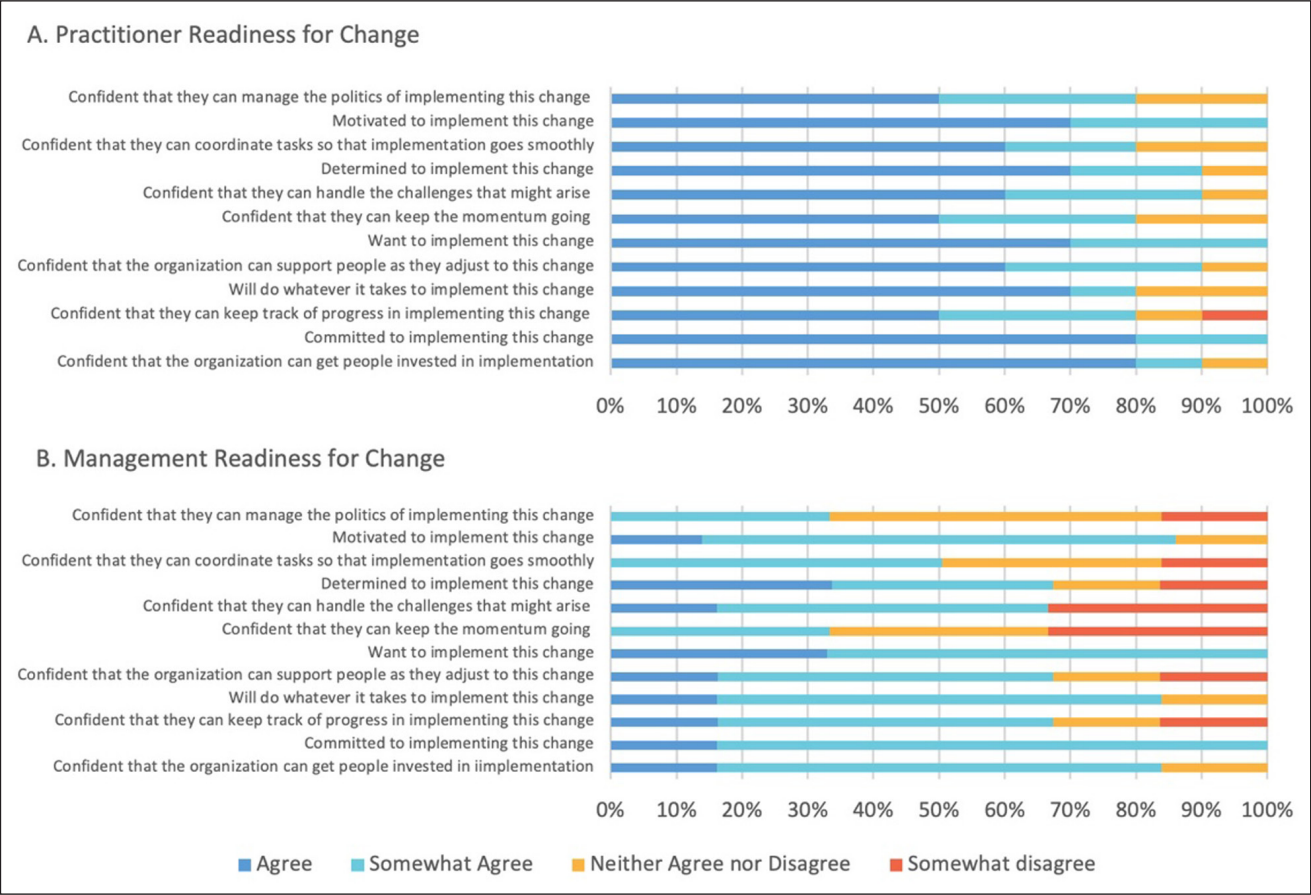
Organisational Readiness for Change.

After the first three months it became clear that there were barriers to practice change, however it was not clear from observational data to what extent organisational barriers to change were impacting on implementation. If organisational readiness for change is low people are more likely to avoid change or even resist taking part in the change process [29]. Shea et al designed a brief measure based on Weiner’s theory of organisation readiness for change called Organisational Readiness for Implementing Change (ORIC) [29]. This tool was used to assess organisational readiness for change across both the practitioners working in the CFH as well as management who formed the CFH advisory group. The ORIC was completed by practitioners (n = 10, 55%) and management (n = 6, 66%) to highlight different barriers for implementation of the CFH as demonstrated in Figure 5. Practitioners were more positive than managers about the implementation of the CFH. Practitioners both wanted to and were committed to implementing the CFH but did not feel confident that they could keep track of progress of implementation. In contrast management were more negative about the overall readiness for change. Managers were committed to implementing the CFH but were not confident that they could keep the momentum going, handle the challenges that might arise or manage the politics of implementation. From observation data, health managers were concerned about the financial investment needed to develop integrated practice. This impacted their confidence of the CFH and may have impacted on their support of their staff. The findings were feedback to CFH practitioners and managers with resulting greater investment and support of the CFH from management.

## Discussion

This case study describes the lessons learned from the implementation of an integrated health and social care CFH and how iteration during implementation supported practice change. At the beginning of the implementation phase, practitioners did not understand the CFH and saw this as a place to make referrals to. It took time for practitioners to fully understand their role in the CFH and to become comfortable with directly identifying and responding to adversity. Practitioners required coaching and additional tools (e.g. postcards and parenting handouts) beyond initial training to achieve the goal of greater identification and referral of clients experiencing adversity. These tools were helpful to both practitioners and families and continued to be used in the maintenance phase of the CFH. The utilisation of emerging innovative qualitative methodologies like rapid ethnography helped support implementation of the CFH. This methodology supported the development of a learning health system approach by the identification of barriers via observation, transcripts from lunchtime learning collaboratives and process data. Iterative changes were then made to training provided, to how the community directory could be accessed and to the development and embedding of referral pathways, further supporting the implementation of the CFH. The changes made during the implementation were then continued and embeded during the maintenance phase of the CFH.

An important lesson learned from the implementation efforts of this CFH was the inclusion of lived experience to help practice change. An interesting recurring barrier during implementation was practitioner discomfort or fear of directly asking about adversity. It was expected that having easier access through the CFH to services would improve practitioner response to adversity however practitioners continued to report fear of causing harm and fear of not being able to respond. This fear and discomfort to ask about social issues is well described with practitioner levels of comfort linked to their personal practice [30,31,32,33,34]. Practitioners find it distressing not to be able to solve problems as described in a paper by Inanici et al *“When I deal with patients who have problems that can’t be solved, I feel emotionally distressed”* [34]. Referral to services may not solve the problems raised by caregivers which drives practitioner behaviour to avoid asking for fear of feeling not adept at fixing problems [35]. The utilisation of lived experience was a strong enabler to overcome practitioner fear and help practitioners to become more willing to try asking directly. Incorporating lived experiences into research and education has been shown to challenge fixed beliefs and to build critically informed understanding while improving connection [36,37]. It can “open the eyes” of the listener to a more wholistic understanding of a person’s experience [36]. Having lived experience involvement both in the codesign of the Hub and then sharing specific lived experiences with practitioners during the learning collaboratives was key to bring about practice change.

The monthly learning collaborative meetings were originally codesigned as case-based discussions [19] however, it became clear that these meetings needed to have a different structure to embed the training and build integrated care practices across health and social care. The meetings were developed into a community of practice which was able to promote learning through social relationships and to co-create solutions to barriers identified during implementation [38]. A key element of the monthly learning collaborative was to have a facilitator who was able to provide coaching and support for practice change. Lau et al. found that facilitation to support education and problem solving within communities of practice has been associated with improved adoption of practice change in primary care [39]. The level of practitioner discomfort and “*overwhelm*” towards the end of our implementation phase may have reflected practitioner concerns of not having this active coaching facilitation role going forward in the CFH.

Practitioners were quick to recognise the benefits of working in a team and developing relationships but understanding of integration was slow to develop. Some practitioners saw their role within the CFH as either making or receiving referrals rather than working in an integrated care practice. This may reflect on the way community health is funded as activity-based funding or fee-for-services has been shown to undermine coordination and integrated care practices [40]. Monthly meetings were an important part of developing integrated care practices however, using financial incentives to improve attendance did not improve practitioner practice change. Financial incentives have been shown to have a positive but variable effect on a range of quality-of-care outcomes in community health [39]. Getting primary care engagement with practice change will likely require a change in how care is delivered and funded in the community, ideally with a move away from a fee-for-service model.

Funding also impacted on how managers perceived the organisational readiness for change. While both practitioners and managers were committed to change, managers did not have the same belief that they could maintain the change in practice. A critical facilitator of practice change is the need for consistent leadership and commitment [41,42]. Ozkalay et al demonstrated the practitioner resistance to change can be attributable to not feeling supported by management [43]. Improving implementation uptake through greater involvement and buy in from management is important however will take time, effort, and financial investment.

Using rapid ethnographic methodology enabled the implementation team to understand the barriers for practitioners and to co-create solutions with practitioners. Having a team approach to implementation whereby observations and field notes are collected by multiple members reduces the risk of bias. Further, identifying barriers as a team through discussion of data ensured credibility of the findings [16]. Ethnographic approaches are especially valuable to studying adaptations to implementation strategies [44]. The implementation of this CFH has shown how implementation underwent several adaptions throughout the first 12 months and how the use of a range of both qualitative and quantitative (process) data was able to guide these changes. Understanding why health care interventions are not taken up is critical if we are to see lasting practice change. This has implications for future implementation projects. We cannot expect to achieve service change through simply training then implementing an intervention if we do not build in opportunities for iteration and use of both qualitative and quantitate data. Using rapid qualitative ethnography provides rapid and actionable insights into overcoming the barriers during implementation and allows for the evolution of interventions into practice.

## Key Learnings

- Practice change takes time and intensive coaching which is generally longer than a research funding cycle.
- Lived experience is a powerful motivator for practice change and is able to overcome practitioner fear of asking about adversity.
- Integration of services requires more than co-location to break down silos.
- Reflective practice is a key driver of practice change with practitioners using reflective practice exercises to iterate personal change.
- Using rapid ethnographic data enables implementation change and directly informed the development of tools aimed at increasing practitioners’ confidence to ask about and respond to adversity.

## Conclusion

Achieving practice change during implementation of an integrated child and family hub requires access to a broad range of data to facilitate iteration and the development of solutions. Investment of adequate funding and time to build relationships is essential for sustained change and functional integration across health and social services.

## Additional File

The additional file for this article can be found as follows:

10.5334/ijic.8631.s1 Supplementary material. Supplementary 1 to 3.
