# Supplementary material for: Lessons Learned From the Implementation of an Integrated Health and Social Care Child and Family Hub – a Case Study
Source: Int J Integr Care. 2024 Nov 15;24(4):9. doi: 10.5334/ijic.8631 (PMC11568806; doi:10.5334/ijic.8631)
Supplement: Supplementary material. — Supplementary 1 to 3. [file ijic-24-4-8631-s1.zip › ijic-8631_loveday/Supplementary 1.pdf]

Ideas for getting more of the behaviour you want and less of the behaviour you don't from your children

Child and Family Hub @Wyndham Vale

'Catch' your child being good

It's not possible to have total control of your child's behaviour.

The best you can do is influence them over time to do more of what you want, and less of what you don't.

Have you noticed?

When a child's doing something we don't want them to, it grabs our attention. We might tell them off, or step in and stop them from doing what they're doing. But, when children are not causing problems, we are less likely to notice what they're doing.

This is not ideal, because children want and need our attention. If they mostly get attention when they're doing the wrong thing, this makes them more likely to do the wrong thing. If they don't get attention for doing the right thing, they're less likely to do the right thing.

You can make a big difference to your child's behaviour just by 'catching' them being good. Being good means doing anything that isn't a problem behaviour. For example, a child sitting at the table doing some colouring is good behaviour because they're not making it hard for those around them to do what they need to. The child is also using skills that may not be obvious at first – they're using their concentration skills to focus; they're using their motor control skills to move their pencil and they're using their creativity to choose different colours.

When you 'catch' your child doing something good (i.e., that isn't a problem), make sure to tell them about it! Praising your child for the kind of behaviour you want to see can make a huge difference in the long term. This can take some practice – check out the examples below if you need some ideas.

"Jason, I love how you're being gentle with your baby sister"

"Minh, you're sitting there so patiently while I talk to the Doctor, well done"

"Mohamed, thank you for asking nicely to play on the iPad"

Maria, you've been so polite letting me finish talking on the phone without interrupting, thank you"

"Abena, you're being so helpful holding the bread for me while we do the shopping"

BETTER BEHAVIOUR

Ideas for getting more of the behaviour you want and less of the behaviour you don't from your children

Child and Family Hub @Wyndham Vale

Quality time

It's not possible to have total control of your child's behaviour.

The best you can do is influence them over time to do more of what you want, and less of what you don't.

Behaviour challenges

Is your child's behaviour causing you problems? If so, you might notice that you're having a lot of negative interactions with your child. You're telling them off, arguing, getting frustrated, and spending a whole lot of time and effort on things that you don't enjoy.

This is very normal in families where children are showing difficult behaviour. Unfortunately, it can leave you and your child feeling bad about your interactions and can make it hard to get better behaviour happening.

Positive moments

When a child's doing something, we don't want them to, it grabs our attention. We might tell them off, or step in and stop them from doing what they're doing. But, when children are not causing problems, we are less likely to notice what they're doing.

This is not ideal, because children want and need our attention. If they mostly get attention when they're doing the wrong thing, this makes them more likely to do the wrong thing. If they don't get attention for doing the right thing, they're less likely to do the right thing.

You can make a big difference to your child's behaviour just by 'catching' them being good. Being good means doing anything that isn't a problem behaviour. For example, a child sitting at the table doing some colouring is good behaviour because they're not making it hard for those around them to do what they need to. The child is also using skills that may not be obvious at first – they're using their concentration skills to focus; they're using their motor control skills to move their pencil and they're using their creativity to choose different colours.

When you 'catch' your child doing something good (i.e., that isn't a problem), make sure to tell them about it! Praising your child for the kind of behaviour you want to see can make a huge difference in the long term. This can take some practice – check out the examples below if you need some ideas.

Cuddle up next to them on the couch and talk about the show they're watching	Pull out some different clothes and play dress-ups	Pull out some toys and each talk about which ones are your favourites
Have a running race around the back yard or up and down your street	Pull out a colouring book and each pick a coloured pencil then work together on a page	Pull out some pots and pans and kitchen utensils and see what different sounds you can make banging them together
