# Supplementary material for: Lessons Learned From the Implementation of an Integrated Health and Social Care Child and Family Hub – a Case Study
Source: Int J Integr Care. 2024 Nov 15;24(4):9. doi: 10.5334/ijic.8631 (PMC11568806; doi:10.5334/ijic.8631)
Supplement: Supplementary material. — Supplementary 1 to 3. [file ijic-24-4-8631-s1.zip › ijic-8631_loveday/Supplementary 2.pdf]

*Practitioner Demographic Data*

| Practitioner Characteristics | N (%)<br>N=18 |
|---|---|
| Age | |
| 18 - 24 years | 1 (5.5) |
| 25 -34 years | 3 (16.7) |
| 35 - 44 years | 7 (38.9) |
| 45 - 54 years | 3 (16.7) |
| 55 - 64 years | 3 (16.7) |
| 65 - 74 years | 1 (5.5) |
| Number of years in role | |
| < 2 years | 2 (11.1) |
| 3 - 5 years | 5 (27.8) |
| 6 - 10 years | 5 (27.8) |
| >10 years | 6 (33.3) |
| Service Provider Gender | |
| Male | 2 (11.1) |
| Female | 16 (88.9) |
| Role | |
| Paediatrician/Paediatric Fellow | 3 (16.7) |
| General Practitioner | 2 (11.1) |
| Nurse (MCHN, Practice Nurse) | 5 (27.8) |
| Allied Health (Speech Pathologist, Dietician) | 2 (11.1) |
| Financial Councillor | 1 (5.5) |
| Lawyer | 3 (16.7) |
| Social worker | 2 (11.1) |
