# Supplementary material for: Lessons Learned From the Implementation of an Integrated Health and Social Care Child and Family Hub – a Case Study
Source: Int J Integr Care. 2024 Nov 15;24(4):9. doi: 10.5334/ijic.8631 (PMC11568806; doi:10.5334/ijic.8631)
Supplement: Supplementary material. — Supplementary 1 to 3. [file ijic-24-4-8631-s1.zip › ijic-8631_loveday/Supplementary 3.docx]

***Coaching Emails sent to Practitioners.***

***Example 1:***

This week, I’ve been thinking about some of the challenges that have come up in our discussions so far. One of these is waiting lists for services and supports. While the lack of accessible services is lamentable, and could be disheartening, what can we do with these families while they wait?

This is a topic that’s touched on by a recent resource published by Emerging Minds. Interestingly, the resource was developed based on input from practitioners in the Barwon region, right next door.

[While you wait: Suggestions for service providers to support children and their families who are on waiting lists - Emerging Minds](https://emergingminds.com.au/resources/while-you-wait-suggestions-for-service-providers-to-support-children-and-their-families-who-are-on-waiting-lists/)

While I don’t think there’s anything revolutionary in the resource, it does invite us to think about what small interactions, pieces of advice, or information, could make a difference to families while they’re waiting for more intensive support. Are there parenting practices they could try out that might make a difference, even if they’re not specifically targeted at the presenting issue? How can we ‘hold’ families as they seek further support?

You may already have ideas along these lines, in which case I encourage you to use them mindfully. If not, I have a strong interest in this area, and would love to meet up to discuss it with you.

All the best and looking forward to seeing all of you next week for our monthly group discussion.

***Example 2:***

Last week we were fortunate to have Leanne come and join us for our monthly case-based discussion meeting, to share some insights from her lived experience of accessing services and talking about adversity. Leanne made some great observations which prompted some valuable discussion.

A key theme which really resonated was:

"Don't underestimate the impact you have in asking about it (adversity)."

Leanne really emphasised that this can make a huge difference to the clients you work with, even if it's not every client who takes up an offer of assistance, or if they take a while to mull it over.

I'm sure you'll agree this is great encouragement to focus on how we 'manage our mandate' to investigate adversity with families. We're continuing to build awareness of the Hub (checkout Teams for the latest), so we're collectively establishing that asking about adversity is a normal and important part of what we do.

If you missed the meeting I’d be more than happy to fill you in, at any time that suits you.
